# Morphological, Physiological, Pathogenic, and Molecular Characterization of *Colletotrichum coccodes* Isolates Associated with Potato Black Dot

**DOI:** 10.3390/plants15182853

**Published:** 2026-09-18

**Authors:** Konstantin S. Troshin, Rashit I. Tarakanov, Peter V. Evseev, Anastasia S. Lysikova, Kirill M. Vorobev, Fevzi S.-U. Dzhalilov

**Affiliations:** 1 Department of Plant Protection, Russian State Agrarian University—Moscow Timiryazev Agricultural Academy, Timiryazevskaya Str. 49, 127434 Moscow, Russia; lysikovanastasia@yandex.ru (A.S.L.); kirillvrbv@mail.ru (K.M.V.); 2 Laboratory of Molecular Microbiology, Pirogov Russian National Research Medical University, Ostrovityanova 1, 117997 Moscow, Russia; petevseev@gmail.com

**Keywords:** *Colletotrichum coccodes*, potato, potato black dot, molecular identification, phylogeny, morphological characteristics, pathogenicity assay

## Abstract

*Colletotrichum coccodes* (Wallr.) S. Hughes is an important causal agent of potato black dot, but variation among isolates collected in Russia remains insufficiently characterized. In this study, 68 isolates obtained from potato tubers and stems collected in major potato-producing regions of Russia and Belarus were characterized using morphological, physiological, pathogenic, multilocus, and whole-genome approaches. The collection showed variation in colony pigmentation and sclerotial distribution, together with statistically significant differences in colony growth rate and lesion diameter in a wounded detached tuber-slice assay. Among the six isolates tested for temperature response, mean growth was highest at 25 °C among the tested temperatures and was strongly reduced at 35 °C. Multilocus sequence analysis of ITS, *act*, *cal*, and *his3* loci identified five haplotypes, revealing limited sequence variation in the four loci analyzed and no obvious geographic pattern in this dataset. Comparative genome analysis of two *C. coccodes* isolates and one *C. nigrum* isolate revealed high genomic similarity between *C. coccodes* genomes, conserved repertoires of secreted proteins, effectors, carbohydrate-active enzymes, cytochrome P450 proteins, and secondary metabolite biosynthetic gene clusters, together with 407 orthogroups present in both sequenced *C. coccodes* isolates and absent from the single *C. nigrum* genome examined. These findings provide a comprehensive characterization of the collection of *C. coccodes* isolates from Russia and show that substantial phenotypic variation within the collection was accompanied by limited sequence variation in the analyzed loci, while representatives of two multilocus haplotypes remained highly similar at the whole-genome level.

## 1. Introduction

*Colletotrichum coccodes* (Wallr.) S. Hughes is the causal agent of potato black dot (also referred to as potato anthracnose), a disease whose economic importance has increased substantially in recent decades. Historically considered of minor significance, it has moved into the category of economically important diseases largely owing to increasing market demands for high-quality washed tubers, both for the fresh pre-packed market and for the processing industry [1,2].

Black dot can cause significant yield losses of up to 30% [3]. Beyond its impact on yield, the disease also leads to considerable weight loss during storage: tubers with black dot symptoms have been reported to lose 5–10% of their fresh weight after 18 weeks of storage, depending on initial disease severity [4]. Furthermore, infection degrades tuber quality through black sclerotial blemishes on the skin and discoloration of internal tissues, substantially reducing marketability. As a result, black dot now ranks among the major constraints to the production of high-quality potatoes in several growing regions worldwide.

Previous studies have documented considerable phenotypic diversity within *C. coccodes* populations, including differences in colony morphology, sclerotial and conidial production, growth rate, aggressiveness, and variability of morphological and physiological characteristics of *Colletotrichum coccodes* isolates in response to the composition of the nutrient medium in vitro [5,6,7,8]. Isolates also differ markedly in aggressiveness on various hosts, including tomato [9], strawberry [10], and potato [8], and host specialization has been reported: for instance, an isolate pathogenic to black nightshade was non-pathogenic to potato [11]. Such differences in aggressiveness within *C. coccodes* populations may partly explain the conflicting results on the effect of the fungus on potato yield reported in the literature [12].

The population structure of *C. coccodes* has been investigated in several parts of the world using vegetative compatibility groups (VCGs). Eight VCGs were identified among isolates from Northern Europe, Israel, France, and the Netherlands [12,13], alongside seven VCGs in North America [14] and six VCGs in Australia [15]. Molecular characterization of *C. coccodes* populations, predominantly based on amplified-fragment length polymorphism (AFLP) analysis, has been performed in North America and Chile [16,17]. By contrast, studies on the population composition of this pathogen in Russia are extremely scarce and have mainly focused on the detection of *C. coccodes* in plant tissue without further comprehensive characterization of the isolates [18,19]. The most detailed molecular study of *C. coccodes* involving Russian material was conducted by Yarmeeva et al. [20]. However, in this study, all *C. coccodes* isolates (originating from potatoes in various regions of Russia, the Netherlands, Germany, Australia, Uganda, and Cyprus) were grouped into a single, well-supported clade in phylogenetic trees based on *act*, *gapdh* and *gs* genes, and no significant genetic polymorphism within the species was detected with the markers used.

In addition, based on phylogenetic analysis using several loci (ITS, *gapdh*, *act*, *tub2*), it was shown that at least two cryptic species, *Colletotrichum coccodes* and *Colletotrichum nigrum* Ellis & Halst, are present on solanaceous crops [20,21]. Because *C. coccodes* and *C. nigrum* are difficult to distinguish morphologically and may not be reliably separated using ITS alone, multilocus sequence analysis is required for accurate species delimitation within this group. Moreover, it was found that *C. nigrum* is also capable of infecting potatoes under experimental conditions [21]. Three isolates from the United States, previously identified as *C. coccodes*, were re-identified as *C. nigrum*. Consequently, many previous studies on the genetic variability of *C. coccodes*, particularly those that relied on ITS markers or did not consider the presence of *C. nigrum*, may contain confounding samples. In the present study, we conducted whole-genome sequencing and comparative analysis of representative isolates of *C. coccodes* and *C. nigrum*.

In this study, we assembled and characterized a collection of 68 *C. coccodes* isolates recovered from potatoes in several production regions of Russia and Belarus. Multilocus sequence analysis of four loci (ITS, *act*, *his3*, and *cal*) was performed to evaluate sequence variation and relationships among the isolates. To extend this comparison beyond the analyzed loci, whole-genome sequencing was performed for two *C. coccodes* isolates representing different multilocus haplotypes and for one isolate of the closely related species *C. nigrum*. Phenotypic characterization included colony morphology and sclerotial arrangement, measurement of conidial and sclerotial dimensions (for 22 isolates), assessment of mycelial growth rate (all isolates), growth response to temperature (6 isolates) and to different nutrient media (17 isolates), and evaluation of aggressiveness on potato tubers.

## 2. Results

### 2.1. Isolation, Colony Morphology and Morphometric Characterization of the Isolates

**Figure 1:**
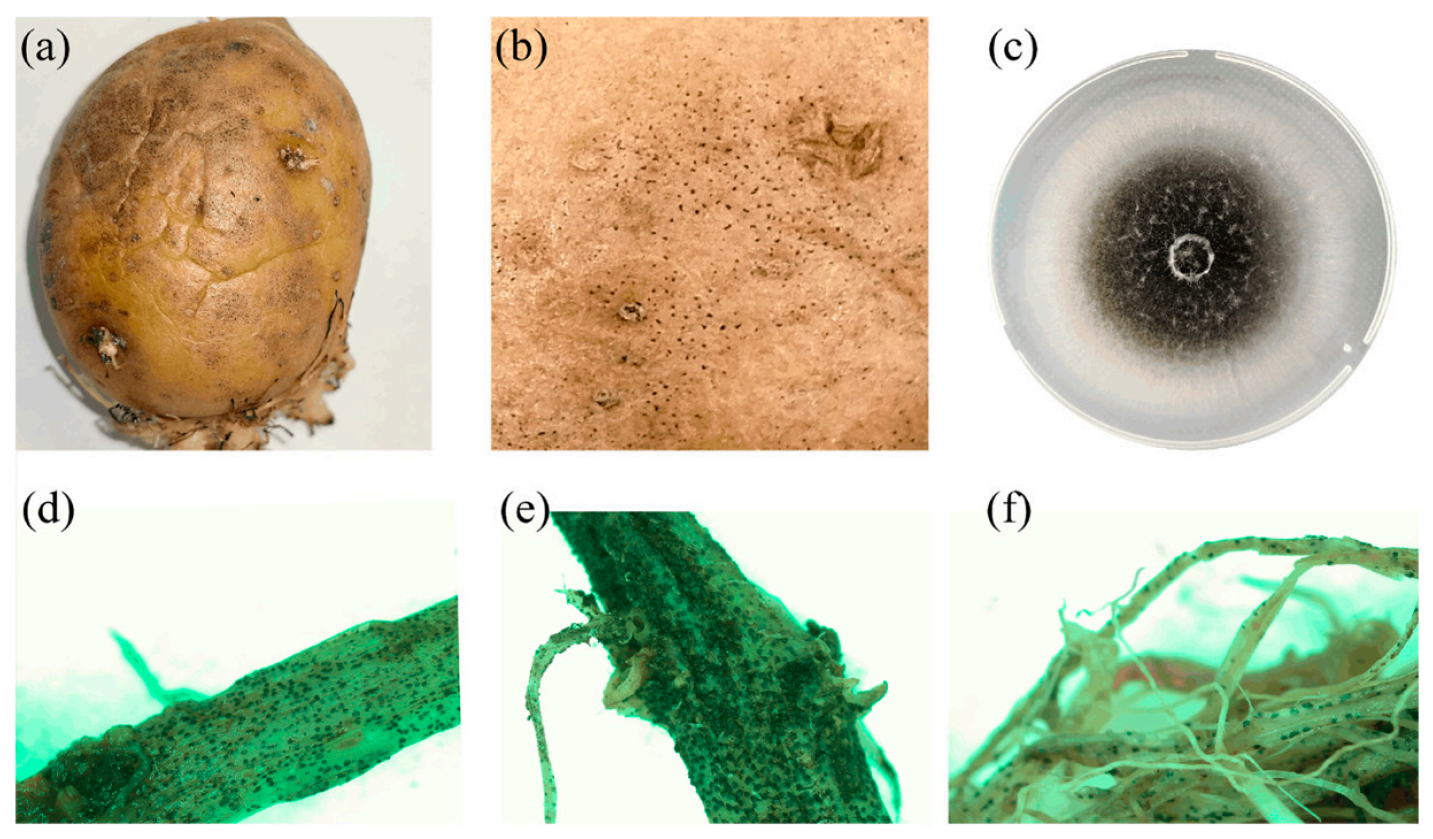
Symptoms of black dot on naturally infected potato tissues collected in this study and a colony of isolated *Colletotrichum coccodes* on PDA. (**a**,**b**) Sclerotia on potato tubers; (**c**) colony on PDA; (**d**,**e**) sclerotia on potato stems; (**f**) sclerotia on potato roots.

Between 2023 and 2026, a total of 65 *Colletotrichum coccodes* isolates were obtained from potato tubers and stems collected in 16 regions of the Russian Federation, and 3 isolates were obtained from the Republic of Belarus (Figure 1, Table S1). Of the 68 isolates, 55 were recovered from potato tubers and 13 from stems.

**Figure 2:**
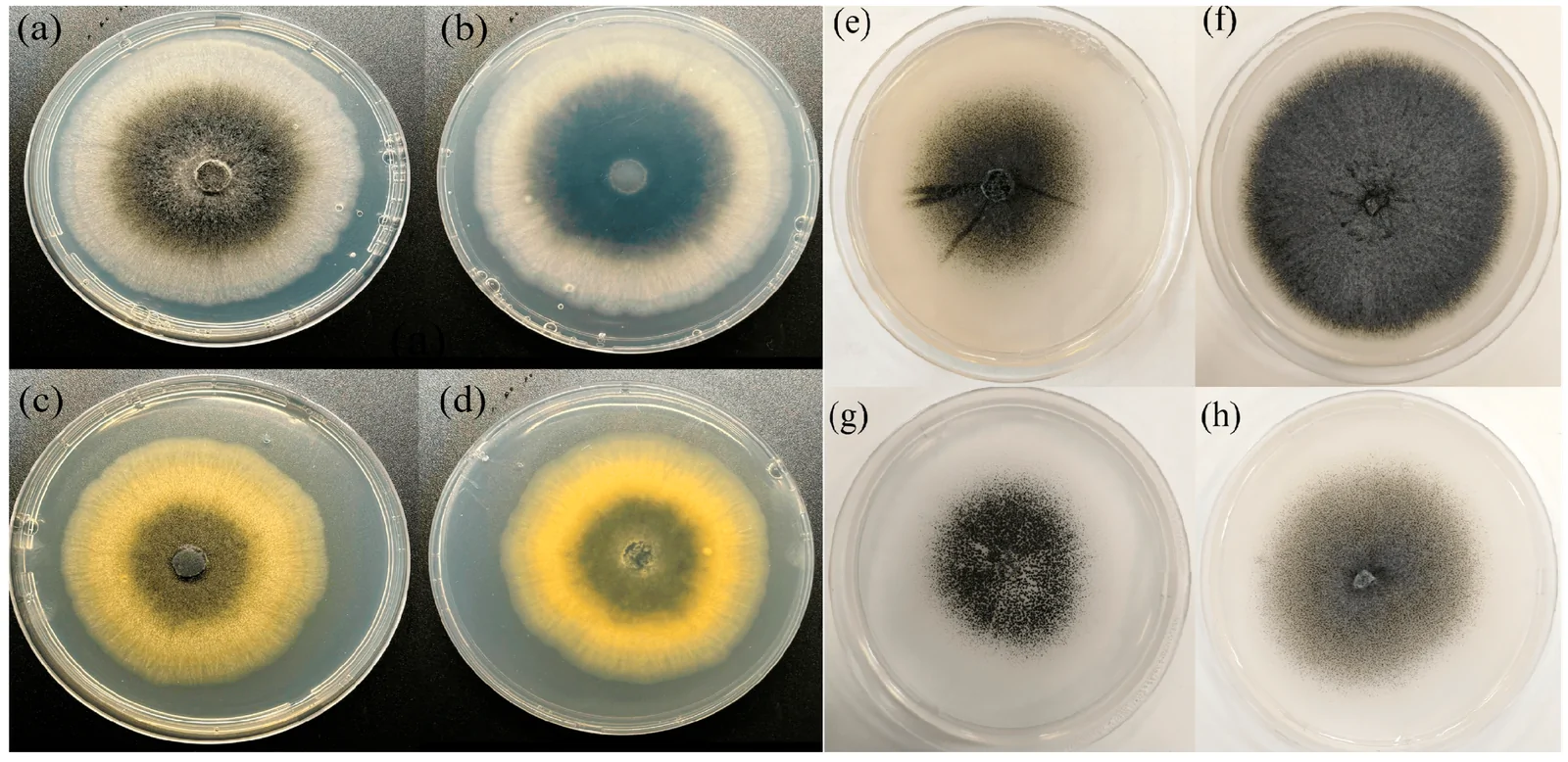
Colony phenotypes and sclerotial morphology groups defined in this study of *Colletotrichum coccodes* isolates grown on potato dextrose agar (PDA) at 25 °C for 7 days. (**a**,**b**) White-to-grey morphotype with a creamy-white to light-orange colony margin; (**c**,**d**) yellow morphotype with more uniform yellow pigmentation. (**e**–**h**) Representative colony morphology groups based on sclerotial density and distribution: (**e**) group A, intermediate sclerotial density with sectors of increased sclerotial production; (**f**) group B, very dense sclerotial formation over the entire colony surface; (**g**) group C, intermediate sclerotial density with sclerotia concentrated mainly near the colony center; and (**h**) group D, sparse and relatively uniform sclerotial distribution.

Based on colony pigmentation, the isolates were classified into two distinct colony color phenotypes: (1) white-to-grey mycelium, occasionally with a creamy-white or pale orange tinge at the colony margin, and (2) yellow colonies. Most isolates had white or grey color, and eight isolates (11.8%) belonged to phenotype 2 (Figure 2).

Colonies were generally flat with sparse aerial mycelium and variable production of black sclerotia. Four qualitative colony morphotypes were distinguished according to the spatial distribution and apparent abundance of sclerotia: A—sectorial variation with alternating areas of lower and higher sclerotial abundance; B—dense sclerotial formation across most of the colony surface; C—predominantly central sclerotial accumulation; and D—sparse sclerotia distributed relatively uniformly across the colony. These categories are descriptive and do not represent measured sclerotial densities. Among the 68 isolates, 6 isolates (8.8%) belonged to group A, 23 (33.8%) to group B, 19 (27.9%) to group C, and 20 (29.4%) to group D (Figure 2).

**Figure 3:**
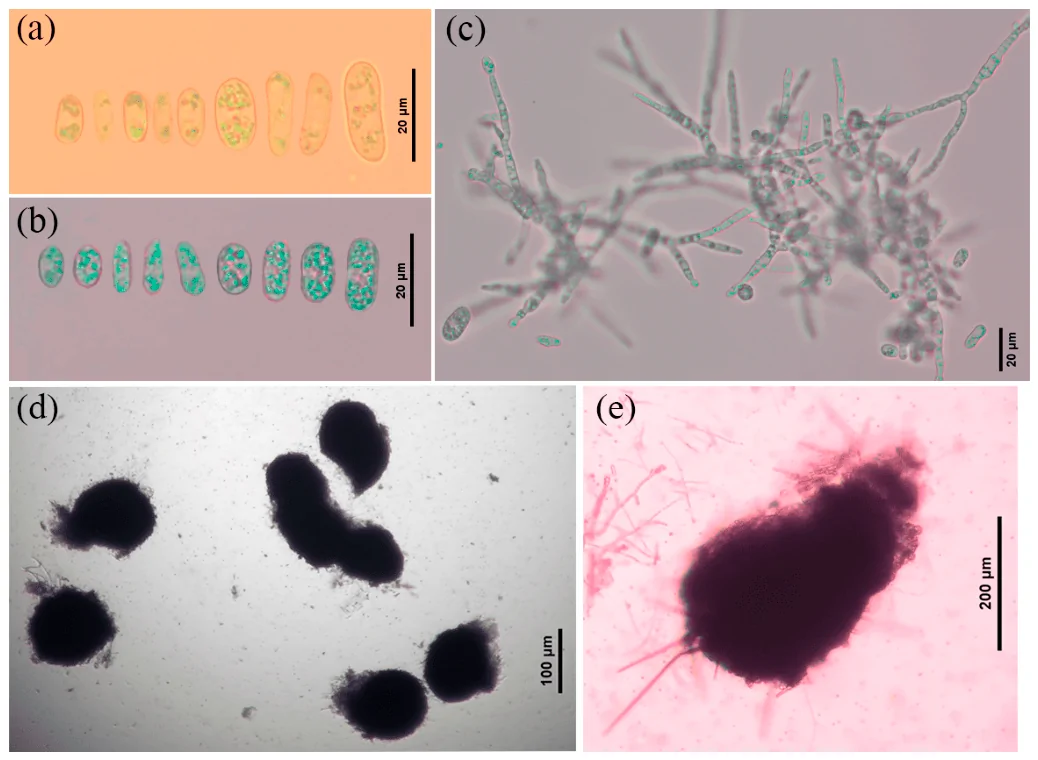
Micromorphological features of representative *Colletotrichum coccodes* isolates cultivated on potato dextrose agar (PDA) at 25 °C for 7 days. (**a**,**b**) Conidia, cylindrical to oval, single-celled; (**c**) mycelium; (**d**,**e**) sclerotia, globose with setae. Scale bars: 20 μm in (**a**–**c**), 100 μm in (**d**), 200 μm in (**e**).

Twenty-two isolates were selected to represent the four morphotypes: five isolates from each of groups A and C and six isolates from each of groups B and D. All examined isolates produced hyaline, aseptate conidia that were cylindrical to ellipsoidal with rounded ends (Figure 3).

Across the twenty-two examined isolates, mean conidial length ranged from 11.7 to 20.7 μm and mean conidial width from 3.5 to 6.3 μm. Mean sclerotial length ranged from 97.0 to 375.0 μm and mean sclerotial width from 73.0 to 290.0 μm. Within-isolate measurements are summarized in Table 1.

### 2.2. Wounded Detached Tuber-Slice Assay and Variation in Aggressiveness and Colony Growth

**Figure 4:**
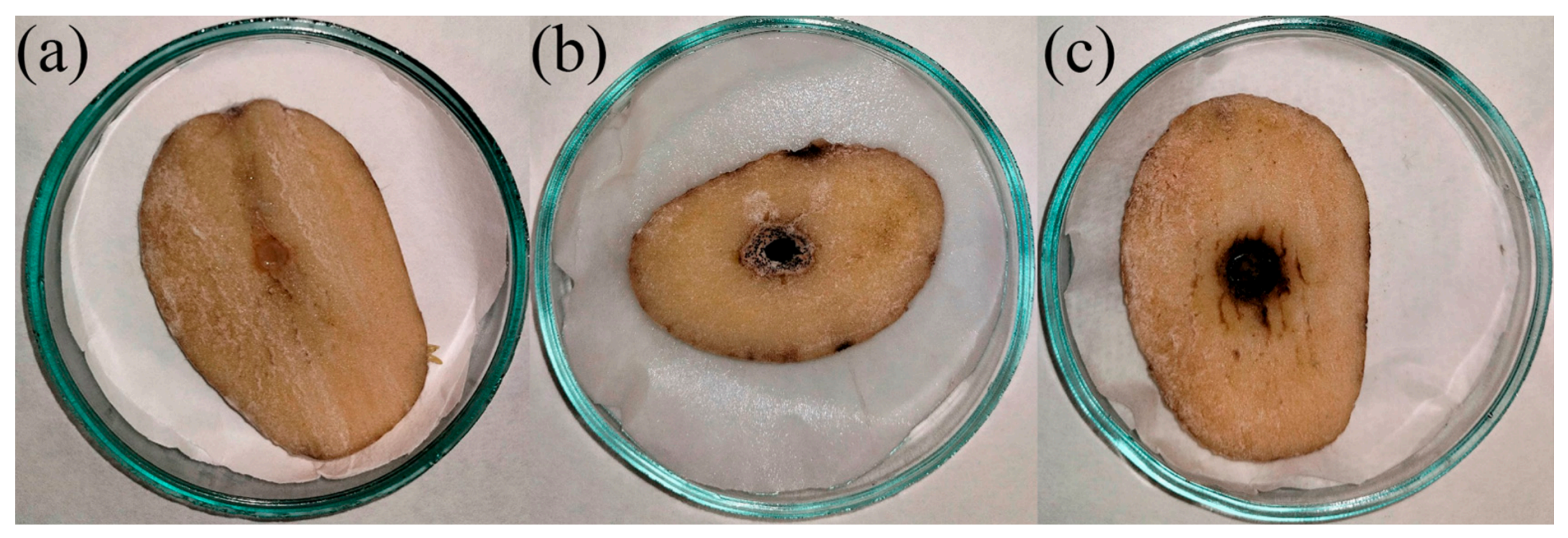
Symptoms produced by *Colletotrichum coccodes* isolates in the wounded detached tuber-slice assay. (**a**) Negative control (potato slice inoculated with sterile agar block); (**b**,**c**) *C. coccodes* Cc47-Lip4 and Cc32-Or3 lesions and sclerotia formed on potato tubers 15 days after inoculation.

All isolates caused visible lesions on potato tuber slices and were successfully re-isolated from symptomatic tissue, whereas no lesions developed in the negative control. Inoculated tuber slices developed brown lesions covered with milky-white mycelium and sclerotia, accompanied by visible tissue maceration beneath the inoculation site. No lesion was observed in the control (Figure 4).

**Figure 5:**
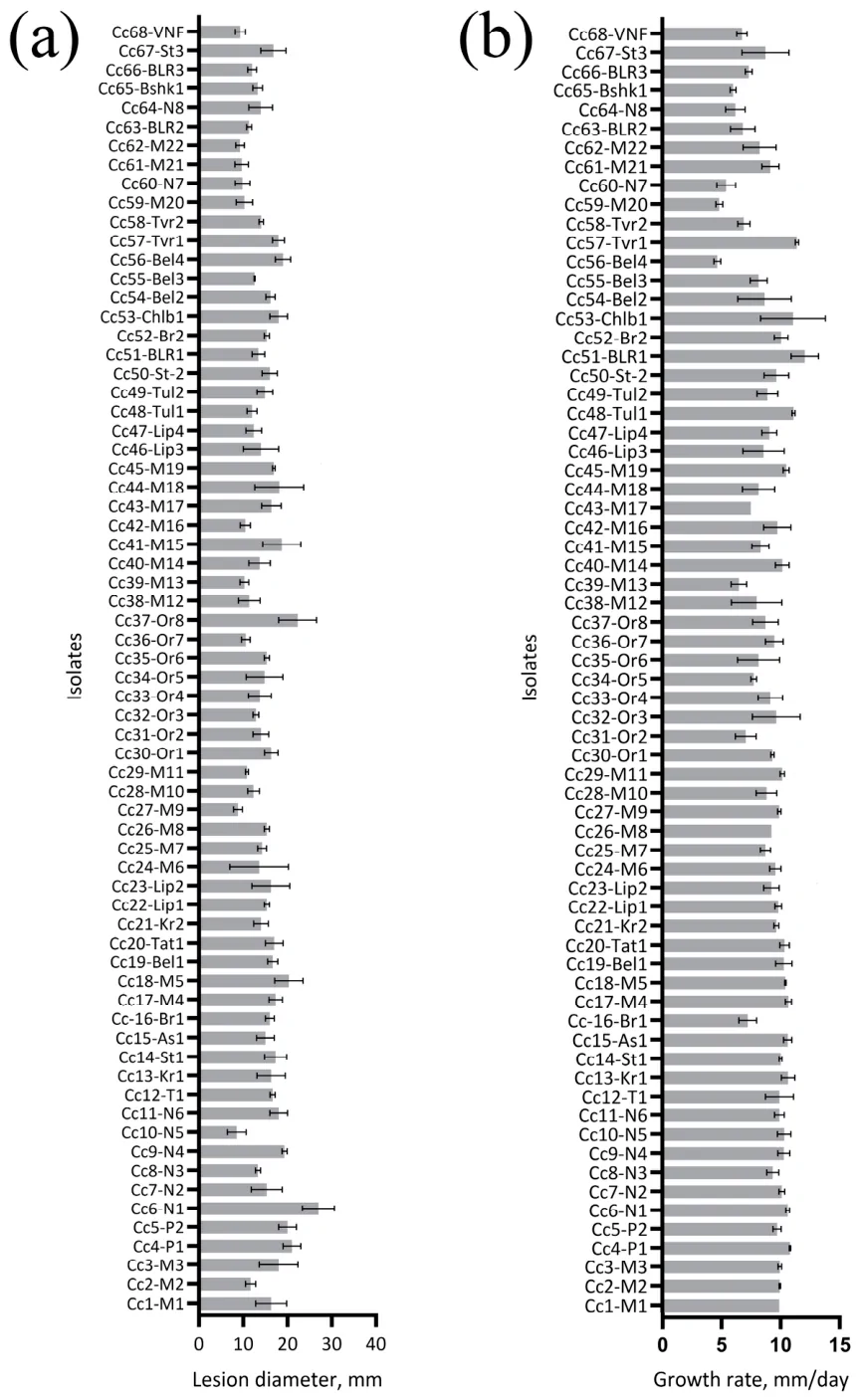
Variation in aggressiveness and mycelial growth rate among *Colletotrichum coccodes* isolates. (**a**) Lesion diameter on potato tubers; (**b**) growth rate on PDA at 25 °C. Bars indicate mean ± SD.

The differences in lesion diameter were statistically significant (*p* < 0.0001). The most aggressive isolate was Cc6-N1 with a lesion diameter of 27 ± 3.6 mm, active growth, and necrosis extending through the full thickness of the tuber slice, while Cc10-N5 showed minimal growth on the potato tuber (8.5 ± 2.1 mm). The overall mean lesion diameter was (mean ± SD) 14.8 ± 3.5 mm (Figure 5).

Growth rates differed significantly among isolates. The highest growth rate was observed for Cc51-BLR1 (12.1 ± 1.16 mm/day), whereas the lowest was observed for Cc56-Bel4 (4.7 ± 0.29 mm/day), *p* < 0.0001 (Figure 5). Spearman’s rank correlation analysis revealed a weak but significant correlation between growth rate and aggressiveness of the isolates, Spearman’s *ρ* = 0.3436, *p* = 0.0041.

### 2.3. Effect of Culture Media on Mycelial Growth

A two-way ANOVA revealed significant main effects of Isolate (*F*(16, 204) = 6.29, *p* < 0.001) and Medium (*F*(5, 204) = 89.5, *p* < 0.001) on growth rate. The significant Isolate × Medium interaction (*F*(80, 204) = 3.3, *p* < 0.001) indicated that differences in growth rate between isolates were not consistent across different nutrient media, and isolates can differ in their physiological responses to medium composition.

Estimation of the effect size through partial *η*^2^ showed a value of 0.69 for the Medium factor, followed by the Isolate × Medium interaction (Partial *η*^2^ = 0.56) and the Isolate factor (Partial *η*^2^ = 0.33).

**Figure 6:**
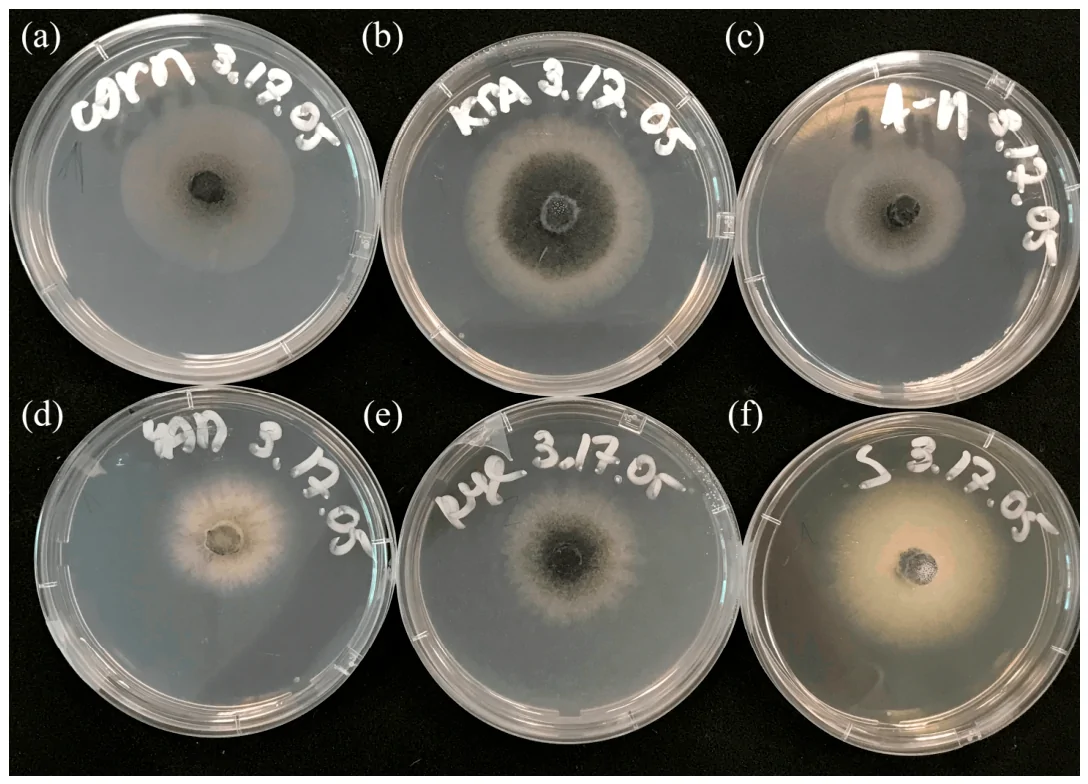
Representative colony appearance of *C. coccodes* isolate Cc17-M4 on six culture media after five days at 25 °C in the dark. (**a**) Corn meal agar; (**b**) potato dextrose agar; (**c**) Asthana & Hawker agar; (**d**) Czapek–Dox agar; (**e**) rye agar; (**f**) Sabouraud dextrose agar.

Descriptively, 10 of 17 isolates showed their highest growth rate on SDA, whereas PDA generally showed the second-highest growth rate. For isolates Cc8-N3, Cc9-N4, Cc12-T1, Cc13-Kr1, Cc15-As1 and Cc17-M4, maximum growth was observed on Czapek–Dox agar. The overall mean growth rates (mean ± SD, *n* = 51, calculated from all isolates and replicates combined for each medium) were: Asthana & Hawker agar 7.8 ± 0.6 mm/day; Czapek–Dox agar 9.2 ± 1.3 mm/day; SDA 10.3 ± 0.8 mm/day; CMA 8.7 ± 0.5 mm/day; Rye agar 8.8 ± 0.8 mm/day; PDA 9.7 ± 0.7 mm/day (Table S2, Figure 6).

The nutrient medium also affected the colony appearance. Colonies on CMA and Asthana & Hawker medium had a smooth surface, even edges, and small sclerotia. On Sabouraud dextrose medium, colonies formed abundant, slimy, shiny masses of conidia during the first 3–4 days of incubation, after which the colony surface wrinkled and sclerotia formed. Colonies on Czapek–Dox medium formed virtually no sclerotia and had a jagged filamentous outer edge. Colonies on rye agar were almost identical to colonies grown on PDA (Figure 6).

### 2.4. Effect of Temperature on Mycelial Growth

**Figure 7:**
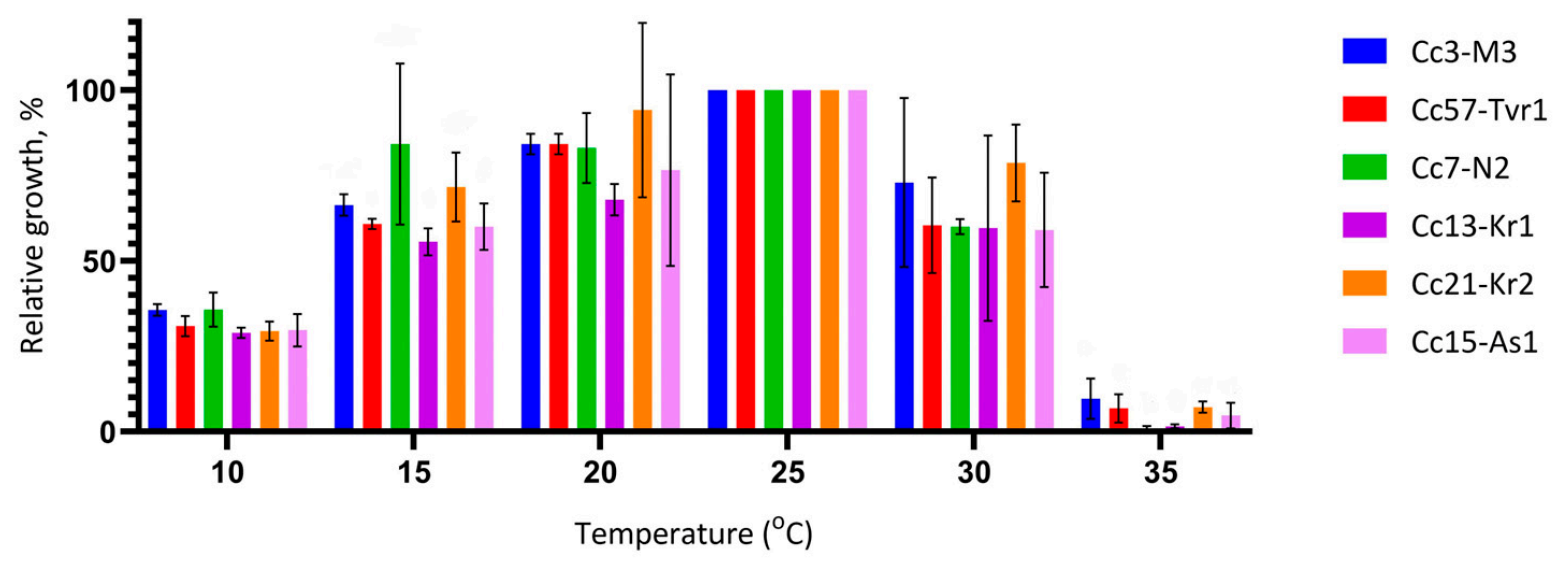
Relative colony diameter growth of six *Colletotrichum coccodes* isolates at different temperatures. Values were normalized to the mean growth rate of each isolate at 25 °C. Bars represent means ± SD of three plates. Statistical inference was based on absolute growth rates.

Across all individual replicate measurements, growth rates ranged from 8.0 to 11.5 mm/day at 25 °C. The highest average growth rate was recorded for isolate Cc15-As1 (from the Astrakhan region), averaging 10.5 mm/day, while the lowest was demonstrated by Cc3-M3 (from the Moscow region), averaging 8.7 mm/day. The relative growth rate, expressed as the growth rate at each temperature relative to that at the optimum temperature (25 °C), varied consistently with temperature (Figure 7).

**Figure 8:**
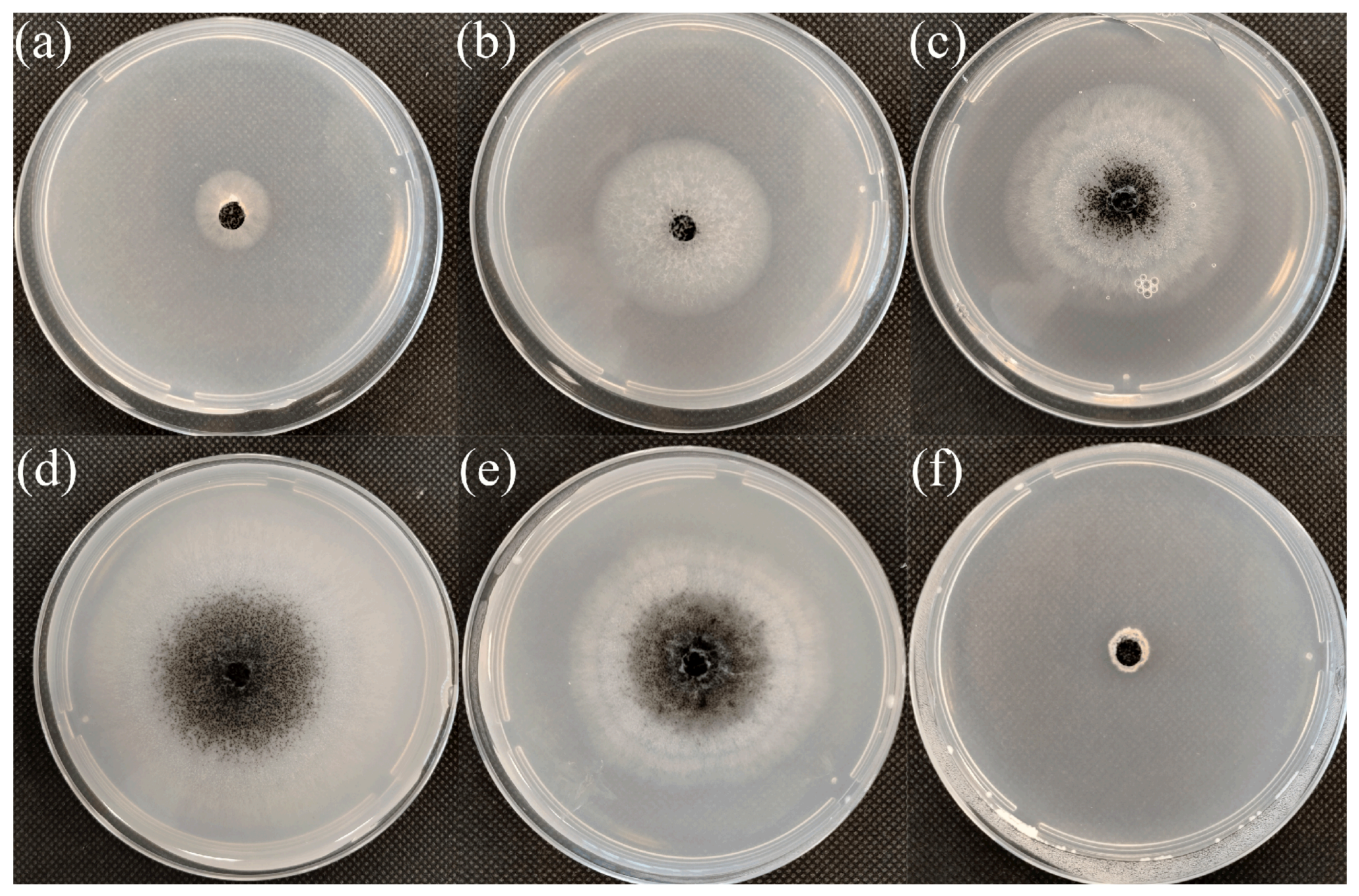
Representative colonies of *C. coccodes* isolate Cc3-M3 on PDA after 5 days of incubation in the dark at: (**a**) 10 °C; (**b**) 15 °C; (**c**) 20 °C; (**d**) 25 °C; (**e**) 30 °C; (**f**) 35 °C.

Two-way ANOVA of absolute colony diameter growth rates detected a temperature effect (F(5, 72) = 216.72, *p* < 0.001), but did not detect an isolate effect (F(5, 72) = 0.42, *p* = 0.83) or an Isolate × Temperature interaction (F(25, 72) = 1.16, *p* = 0.31). The highest overall mean growth rate among the tested temperatures occurred at 25 °C, whereas growth was strongly reduced at 35 °C (Figure 8).

### 2.5. Multilocus SNP Haplotypes, Species-Level Phylogenetic Placement, and Comparative Genomics

MLST analysis was performed to determine whether the 68 *Colletotrichum coccodes* isolates could be divided into sequence groups. The final four-locus concatenated alignment included *act*, *cal*, *his3*, and ITS. Five polymorphic positions were retained, all located in *cal*, *his3*, and ITS, whereas *act* was invariant among the analyzed isolates. The variable positions were CAL:37, CAL:117, HIS3:230, HIS3:360, and ITS:449. Their combinations defined five haplotypes: H5 included 25 isolates, H2 included 19, H1 included 16, H4 included seven, and H3 was represented by one isolate (Supplementary Table S3).

**Figure 9:**
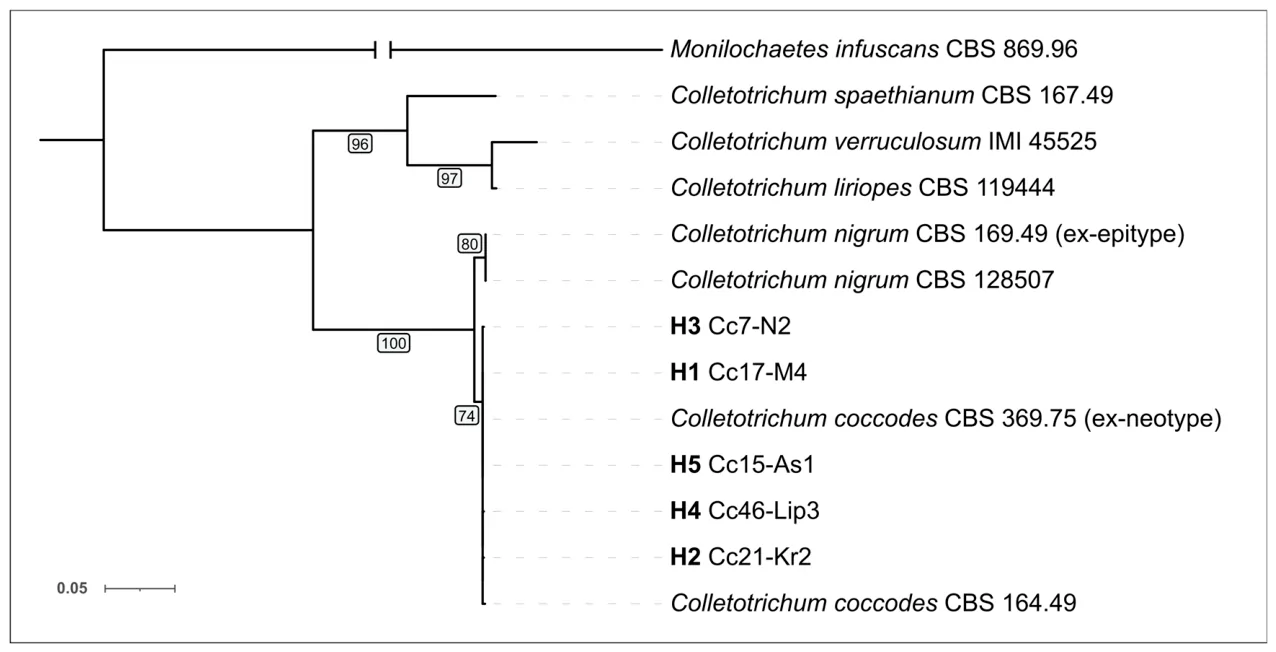
Partitioned maximum-likelihood phylogeny of representative *Colletotrichum coccodes* haplotypes and type-derived/reference strains based on concatenated *act*, *his3*, and ITS sequences. The alignment comprised 1128 nucleotide positions, including 237 bp of act, 373 bp of *his3*, and 518 bp of ITS. H1–H5 represent isolates selected from the five four-locus SNP haplotypes identified among the 68 *C. coccodes* isolates analyzed in this study. *C. coccodes* CBS 369.75 is an ex-neotype culture, and *C. nigrum* CBS 169.49 is an ex-epitype culture. *Monilochaetes infuscans* CBS 869.96 was used as the outgroup. Numbers at internal nodes indicate ultrafast bootstrap support (%) based on 1000 replicates; values below 70% are not shown. The branch leading to the outgroup was graphically shortened for visualization. The scale bar indicates substitutions per site.

Species-level phylogenetic placement was assessed separately using concatenated *act*, *his3*, and ITS sequences from one representative of each of the five four-locus haplotypes together with type-derived and reference strains of *C. coccodes*, *C. nigrum*, and related taxa (Figure 9). The three-locus alignment comprised 1128 nucleotide positions, including 284 variable and 109 parsimony-informative sites. All five haplotype representatives grouped with *C. coccodes* CBS 369.75 (ex-neotype) and CBS 164.49 (UFBoot = 74%), whereas *C. nigrum* CBS 169.49 (ex-epitype) and CBS 128507 formed a separate clade (UFBoot = 80%). Among the haplotype representatives, H1 and H5 were identical to CBS 369.75 across the aligned *act*, *his3*, and ITS regions. Relationships among the *C. coccodes* haplotype representatives were poorly resolved, consistent with the limited sequence variation within the collection.

The two genome-sequenced *C. coccodes* isolates represented different multilocus haplotypes: strain 17-M4 was assigned to H1, whereas 46-Lip3 was assigned to H4. These haplotypes differed at CAL:37, CAL:117, and ITS:449. Whole-genome ANI showed that 17-M4 and 46-Lip3 were nevertheless closely related. Their symmetric mean ANI was 99.5229%. *C. nigrum* C21KSPeF19 showed ANI values of 96.8197% to 17-M4 and 96.7889% to 46-Lip3. The pairwise comparisons separated *C. nigrum* C21KSPeF19 from the two *C. coccodes* isolates, while confirming the close nucleotide-level relationship among all three genomes.

The three draft genome assemblies ranged from 49.55 to 51.35 Mb and comprised 650–1460 contigs (≥200 bp), with N50 values of 186.6–536.1 kb. BUSCO analysis in genome mode indicated high assembly completeness (99.4–99.5%; Supplementary Table S4). The genome annotations were comparable in completeness and gene content. BUSCO completeness of the final predicted protein sets was 98.1% for 17-M4, 98.1% for 46-Lip3, and 97.5% for C21KSPeF19.

The respective annotations contained 12,409, 12,425, and 12,552 CDSs. Hypothetical proteins accounted for 76.59%, 76.68%, and 76.86% of the predicted CDSs, respectively. The numbers of predicted secreted proteins, effectors, and CYP450-like proteins were also similar. DeepTMHMM assigned 1537 proteins in 17-M4, 1550 in 46-Lip3, and 1525 in *C. nigrum* C21KSPeF19 to the SP class; these sequences constituted the predicted secretomes. EffectorP analysis of these predicted secretomes identified 466, 471, and 470 candidate effectors, respectively. The corresponding numbers of CYP450-like proteins were 220, 220, and 219.

The CAZyme repertoires differed little among the genomes. dbCAN assigned 2536, 2543, and 2529 proteins to CAZyme families in *C. coccodes* Cc17-M4, *C. coccodes* Cc46-Lip3, and *C. nigrum* C21KSPeF19, respectively, when assignments supported by at least one dbCAN component were retained. The corresponding high-confidence sets supported by at least two dbCAN tools comprised 794, 795, and 789 proteins. Intersection of the broader CAZyme-family-assigned sets with the DeepTMHMM SP secretomes identified 615, 622, and 610 predicted secreted CAZymes, respectively. No CAZyme family was present in both *C. coccodes* genomes and absent from *C. nigrum* C21KSPeF19 in either the broader CAZyme set or the high-confidence subset supported by at least two dbCAN tools. AntiSMASH predicted 80 regions in *C. coccodes* Cc17-M4, 84 in *C. coccodes* Cc46-Lip3, and 80 in *C. nigrum* C21KSPeF19. Because individual regions could receive more than one product designation, these regions corresponded to 101, 103, and 105 product-class assignments, respectively. All three genomes contained the same major predicted product classes: T1PKS, NRPS, NRPS-like, terpene, indole, T3PKS, terpene-precursor, betalactone, and isocyanide. *C. nigrum* C21KSPeF19 had slightly more NRPS, T1PKS, indole, and terpene product-class assignments, whereas the two *C. coccodes* genomes had more terpene-precursor and T3PKS product-class assignments. These differences did not indicate the gain or loss of a major predicted biosynthetic product class.

The orthogroup analysis provided the clearest distinction. OrthoFinder identified 11,624 orthogroups shared by all three genomes, including 11,245 single-copy orthogroups. A further 407 orthogroups were shared by *C. coccodes* 17-M4 and 46-Lip3 but were absent from *C. nigrum* C21KSPeF19. They contained 827 genes, including 413 from 17-M4 and 414 from 46-Lip3. In comparison, 15 orthogroups containing 40 genes were present only in *C. nigrum* C21KSPeF19 within the three-genome comparison. No orthogroups were unique to only one of the two *C. coccodes* genomes. Gene-level annotations for all 407 candidate orthogroups, including the 78 priority orthogroups selected for closer examination, are provided in Supplementary Table S6.

The 407 *C. coccodes*-associated orthogroups contained 96 predicted secreted proteins, 48 EffectorP-positive candidates, 37 CAZyme genes, 22 CAZyme genes supported by at least two dbCAN tools, and 29 CYP450-like genes. Seventy-eight orthogroups were selected for further examination: 24 encoded predicted secreted effectors, 21 encoded other secreted proteins, 14 contained CYP450-like proteins, four contained secreted CAZymes, and two contained secreted EffectorP-positive CAZymes. The latter included a CE8/pectinesterase-related orthogroup with pectin lyase-fold and pectinesterase catalytic signatures and an AA7/FAD-linked oxidoreductase-like orthogroup.

## 3. Discussion

Potato black dot was first reported in the nineteenth century, and the first detailed description of the disease was published in 1926. Since then, the importance of the disease has greatly increased due to the demand for high-quality tubers for the fresh pre-packed market and a growing body of evidence demonstrating its negative effect on yield [1,2,3,4].

In Russia, *C. coccodes* was first discovered in 1965; potato black dot was detected in some batches of potatoes received from the Smolensk, Novgorod, Bryansk, Kostroma, Pskov and Leningrad regions, with significant yield losses reported in the Leningrad region [22]. The occurrence of black dot in Russia has increased in subsequent years. In recent studies, *C. coccodes* was found in potato samples from North Ossetia, Kostroma region, the Mariy El Republic [19], Vladimir region, Primorsky Krai, and the Republic of Tatarstan [20]. The management of the black dot is complicated by several factors. Firstly, the symptoms can be easily mistaken for other diseases, such as silver scurf, so farmers may fail to apply appropriate control measures. Secondly, sclerotia of *C. coccodes* can survive in soil for up to 13 years in the absence of potato [23,24] and can infect a wide range of weeds and rotation crops [25]. Finally, the Russian State Standard for seed potatoes (GOST 33996-2016 [26] and GOST R 59551-2021 [27]) does not directly restrict the levels of potato black dot infection, leading to circulation of infected seed material within the country. GOST R 59551-2021 (in Section 4.2.2, Table 3 of the standard) only indirectly addresses potato black dot through general limits on shriveled or damaged tubers, and GOST 33996-2016 contains no provisions regarding black dot at all. Collectively, these factors make black dot an economically important disease for potato production in Russia and highlight the need for improved monitoring and management strategies.

The obtained *C. coccodes* isolates showed substantial variability in morphological characteristics. Four morphological groups were determined based on sclerotial density and arrangement, with group B being the largest. Some similar groups have also been described in other studies. Rodeva et al. [28] observed colonies with concentric rings of sclerotia and colonies with radial sclerotial stripes, which, in our study, corresponded to the smallest morphology group A; however, no isolates with concentric rings on PDA were observed in our collection. Most isolates had white or grey mycelium with a creamy white or pinkish outer layer, which was also reported by Rodeva et al. [28]. Eight isolates exhibited yellow colony pigmentation after several passages under the culture conditions used in this study. This was recorded as a descriptive phenotype. To our knowledge, stable yellow colony pigmentation has rarely been documented for *C. coccodes* and may represent an uncommon phenotypic variant. Standard morphological descriptions of *C. coccodes* indicate the formation of white or grayish mycelium [6,28,29,30]. The sizes of sclerotia and conidia of our isolates were consistent with the species description of *C. coccodes*, despite the known variability of these characteristics, which may depend on factors such as the composition of the nutrient media and the VCG of the isolate [8].

Because the number of isolates varied considerably among regions, the present collection was not designed for formal testing of geographic structure. Geographic comparisons should therefore be interpreted descriptively.

The results of the aggressiveness and growth rate tests showed that all isolates (stem- and tuber-derived) colonized the wounded detached potato tuber slices, producing necrotic lesions under controlled conditions and differed significantly in lesion diameter and growth rate on PDA, confirming the presence of substantial diversity in aggressiveness of isolates within the present isolate collection. Growth rate on PDA and lesion diameter were only weakly associated. These assays measure different aspects of fungal performance: colony expansion on an artificial medium and lesion development in wounded host tissue. Host-interaction traits and extracellular metabolism may contribute to lesion development [31,32,33], but these mechanisms were not measured in this study.

The significant Isolate × Medium interaction (*p* < 0.001) indicates that differences in growth rate among isolates are not consistent across culture media, i.e., the physiological response to nutrient composition varied among isolates within the presented *C. coccodes* collection. The Isolate × Medium interaction had a substantial partial effect size (Partial *η*^2^ = 0.56), suggesting that medium-dependent growth responses represent a meaningful component of diversity in this collection. For example, Aqeel et al. [8] reported that the growth response to different culture media varied among NA-VCGs of *C. coccodes*, indicating that physiological variation exists among vegetative compatibility groups as well as among individual isolates.

Qualitative differences in colony morphology on different media were also observed. On Czapek–Dox agar, sclerotial formation was almost completely absent, and colonies developed a jagged filamentous margin, while on CMA and Asthana & Hawker agar, sclerotia were sparse and the colony margin was smooth. On Sabouraud dextrose agar, abundant conidial masses were produced during early incubation. These observations are consistent with the known influence of nutrient composition on mycelial growth, sporulation, and sclerotial differentiation in *Colletotrichum* spp. [8,34].

The six isolates tested across a temperature range of 10 to 35 °C showed the highest overall mean growth at 25 °C, and strong inhibition at 35 °C. Glais-Varlet et al. [35] reported the fastest growth at 27 °C on malt agar over a tested range of 5–27 °C. Differences in medium and temperature intervals should therefore be considered when comparing reported growth optima.

The factorial analysis did not detect an Isolate effect or an Isolate × Temperature interaction. This does not establish equivalence among isolates or exclude geographic variation. The temperature comparison was limited to six isolates.

Five variable positions in the MLST loci defined five haplotypes among the 68 *C. coccodes* isolates, revealing limited intraspecific variation. The haplotypes showed no obvious geographic segregation, although uneven regional sampling precluded a formal assessment of geographic structure. The small number of polymorphic sites also limited the resolution of the haplotype scheme. ANI between the two *C. coccodes* genomes was 99.5229%, compared with approximately 96.8% between *C. nigrum* C21KSPeF19 and either *C. coccodes* isolate. The 95–96% ANI boundary commonly used for prokaryotes [36] provides a useful reference but cannot be applied directly to fungi. Genome-based criteria for fungal species delimitation remain lineage-dependent, although ANI and related measures can distinguish closely related fungal species when calibrated within a particular group [37,38]. The values obtained here show that *C. nigrum* C21KSPeF19 is more distant from the two *C. coccodes* genomes than these genomes are from each other, but do not constitute an independent species criterion.

Despite their assignment to different multilocus haplotypes, the two *C. coccodes* isolates were highly similar in nucleotide sequence, gene number, and the broad composition of their predicted secretome, effector, CAZyme, CYP450, and secondary-metabolite repertoires. The limited number of sequenced isolates does not allow the genetic basis of the phenotypic variation observed across the collection to be determined. Nevertheless, the comparison shows that representatives of two multilocus haplotypes remain closely related at the whole-genome level.

The corresponding functional repertoires of *C. nigrum* C21KSPeF19 were also broadly similar, consistent with the close relationship and limited morphological differentiation between *C. coccodes* and *C. nigrum*. Both species can cause lesions on potato tissues in experimental assays; in particular, one *C. nigrum* isolate produced lesions on detached potato leaves within the range observed for *C. coccodes*, although its importance as a potato pathogen under field conditions remains unclear [20,21]. The clearest gene-content difference was the presence of 407 orthogroups in both *C. coccodes* genomes and their absence from *C. nigrum* C21KSPeF19. These orthogroups included secreted proteins, predicted effectors, CAZymes, and CYP450-like proteins. Differences in these gene groups can contribute to host interaction and host specificity in *Colletotrichum* [39]. However, the comparison included only two *C. coccodes* genomes and one *C. nigrum* genome. The 407 orthogroups should therefore be considered candidate *C. coccodes*-associated genes rather than species-specific genes.

## 4. Materials and Methods

### 4.1. Isolation and Preservation of Fungal Isolates

Potato tubers and stems with typical symptoms of anthracnose were collected from commercial fields with the aid of the Syngenta Laboratory of Technical Support, Moscow, Russia. Plant material was obtained from 16 regions of the Russian Federation and 1 region of the Republic of Belarus. To avoid repeated sampling from the same plant material, only one isolate was retained from each individual tuber or stem sample. Samples were obtained opportunistically. Exact field-level provenance was not consistently recorded, and possible clustering of isolates within fields could not be assessed. The collection was therefore not treated as a geographically representative population sample.

To isolate *Colletotrichum coccodes*, tubers were washed in a 1% (*v*/*v*) aqueous solution of Domestos household bleach (Arnest UniRus LLC, Moscow, Russia), prepared by diluting the commercial product containing 2.7–3.3% available chlorine with tap water. The tubers were then rinsed with tap water to remove residual bleach and surface dirt and dried on filter paper. Tuber pieces with microsclerotia (1 × 1 cm) were cut with a sterile scalpel and surface-sterilized for 1 min in 10% Domestos household bleach solution, followed by 30 s in 70% ethanol, rinsed three times with sterile distilled water (SDW), dried on a sterile filter paper and placed on potato dextrose agar (200 g potato, 20 g dextrose, 15 g agar per 1 L) supplemented with antibiotics (streptomycin sulfate and chloramphenicol (Central Drug House (P) Ltd., Delhi, India), each at 100 mg/L) to suppress bacterial contamination. Stems were first cut into pieces 0.5 cm in length and rinsed under running tap water for 30 min then surface sterilized and plated as described above. All plates were incubated in the dark at 25 °C for 3 to 5 days.

*C. coccodes* colonies were identified preliminarily based on their morphology under a Stemi 508 microscope (Zeiss, Oberkochen, Germany). An agar disk from the edge of a colony with actively growing mycelium was transferred to a new PDA plate with a microbiological needle.

To obtain monoconidial cultures, a plate with 7-day-old culture was flooded with 5 mL of SDW and scraped with a sterile loop, and the resulting conidial suspension was filtered through four layers of sterile cheesecloth. The filtered suspension was streak-plated with a loop, and after 24–72 h of incubation, germinated single conidia or well-separated single-conidium colonies were transferred to fresh PDA plates.

Isolates were stored in two ways: at −80 °C in 25% (*v*/*v*) glycerol/SDW solution for long-term and at 4 °C in SDW for short-term preservation (for up to three months).

### 4.2. DNA Extraction, PCR Amplification, and Sequencing

For DNA extraction, mycelium with sclerotia from 7-day-old cultures were carefully scraped from the agar surface, transferred into 2 mL tubes with steel beads and homogenized using Bioprep 24R homogenizer (Hangzhou Allsheng Instruments Co., Ltd., Hangzhou, China). The resulting homogeneous suspension was centrifuged for 1 min at 1700× *g* (MiniSpin, Eppendorf, Hamburg, Germany), and the supernatant was taken for DNA extraction using Allsheng Auto-Pure 96 Nucleic Purification System (Hangzhou Allsheng Instruments Co., Ltd., Hangzhou, China) with a Phytoscreen-Express commercial DNA extraction kit (SYNTOL, Moscow, Russia).

The isolated DNA was used to amplify the internal transcribed spacer region (ITS, ITS1-5.8S-ITS2) with primers ITS1 and ITS4 [20,40], *act* with primers ACT-512F and ACT-783R [41], *cal* with primers CL1C and CL2C [42,43], and *his3* with primers CYLH3F and CYLH3R [44] (Table 2).

Amplifications of ITS, *act*, *cal* and *his3* were performed in 0.2 mL tubes (Eppendorf, Hamburg, Germany) in a 25 μL total volume reaction containing 1 μL of a DNA template (40 ng/μL), 5 μL of 5× master mix (Dialat LTD, Moscow, Russia), 0.4 μL of each primer (10 μM) and 18.2 μL of ddH_2_O.

The PCR protocol included initial denaturation at 94 °C for 3 min, 35 amplification cycles, and an additional extending step at 72 °C for 5 min. For the primer pair ITS1/ITS4, the amplification cycles were 94 °C for 30 s, 55 °C for 30 s, and 72 °C for 45 s. For the primer pair ACT-512F/ACT-783R, the amplification cycles were 94 °C for 30 s, 67 °C for 30 s and 72 °C for 45 s. The amplification parameters for the primer pair CL1C/ CL2C were 94 °C for 40 s, 57 °C for 40 s and 72 °C for 1 min. The amplification parameters for the primer pair CYLH3F/CYLH3R were 94 °C for 30 s, 52 °C for 30 s and 72 °C for 30 s.

The amplification was performed on a T100 Thermal Cycler (Bio-Rad Laboratories, Inc., Hercules, CA, USA). PCR products were run in 1.5% agarose gel containing ethidium bromide. The PCR product was purified using the ColGen commercial kit (SYNTOL, Moscow, Russia) prior to sequencing. Sanger sequencing was carried out by Evrogen Co., Moscow, Russia.

### 4.3. Multilocus SNP Haplotype Analysis and Species-Level Phylogenetic Placement

Forward and reverse Sanger reads were manually inspected and curated in Geneious Prime 2025.0.3 (Biomatters, Inc., Auckland, New Zealand), and consensus sequences were generated for the *act*, *cal*, *his3*, and ITS loci. For the within-collection haplotype analysis, 20 terminal positions were trimmed from each end of each locus alignment, after which columns containing at least one gap were removed. The resulting four-locus alignment comprised 1872 nucleotide positions across the 68 study isolates and the *Colletotrichum coccodes* reference strain CBS 369.75, including 235 bp of act, 740 bp of *cal*, 373 bp of *his3*, and 524 bp of ITS. SNP haplotypes were defined from variable nucleotide positions among the 68 study isolates. SNP coordinates were numbered relative to the ungapped sequences of the corresponding loci of *C. coccodes* CBS 369.75. Isolates with identical nucleotide states at all variable positions were assigned to the same SNP haplotype.

Species-level phylogenetic placement was assessed separately using *act*, *his3*, and ITS, which constituted the common set of loci available for all selected taxa. One isolate from each of the five four-locus haplotypes was included: Cc17-M4 (H1), Cc21-Kr2 (H2), Cc7-N2 (H3), Cc46-Lip3 (H4), and Cc15-As1 (H5). Reference sequences of *C. coccodes*, *C. nigrum*, related *Colletotrichum* species, and the outgroup were retrieved from GenBank; strain information and accession numbers are provided in Supplementary Table S7. Each locus was aligned separately with MAFFT v7.526 using the --auto option [45]. The alignments were visually inspected in Geneious Prime, and terminal columns outside the region covered by all sequences were removed; internal alignment gaps were retained. The resulting alignments comprised 237 bp of *act*, 373 bp of *his3*, and 518 bp of ITS and were concatenated into a 1128 bp alignment containing 13 sequences.

A partitioned maximum-likelihood phylogeny was inferred with IQ-TREE v3.0.1 [46], with *act*, *his3*, and ITS treated as separate partitions under an edge-linked proportional partition model. ModelFinder [47] selected the best-fit substitution model for each partition according to the Bayesian information criterion (BIC): TN+G4 for *act*, TIM3+F+I+G4 for *his3*, and TNe for ITS. Branch support was assessed using 1000 ultrafast bootstrap and 1000 SH-aLRT replicates. *Monilochaetes infuscans* CBS 869.96 was specified as the outgroup, and the tree was visualized in iTOL [48].

### 4.4. Genome Sequencing and Analysis

Whole-genome paired-end sequencing of the selected *Colletotrichum coccodes* isolates Cc17-M4 and Cc46-Lip3, representing H1 and H4 haplotypes, and one *Colletotrichum nigrum* isolate C21KSPeF19 [20], obtained from the collection of Dr. Elansky, was performed in PE125 mode on the DNBSEQ-G400 platform (MGI Tech Co., Ltd., Shenzhen, China).

De novo genome assembly was performed using SPAdes v4.0.0 [49]. Assembly statistics, including total length, number of contigs, N50, and GC content, were calculated with QUAST v5.3.0 [50]. Nominal sequencing depth was calculated as the total number of bases in the raw paired-end reads divided by the final assembly size. Contamination screening was performed at the contig level. Assembled contigs were taxonomically classified with Kraken2 v2.1.1 [51]. Contigs assigned to bacterial taxa were removed. MetaBAT2 v2.18 was used for binning-based inspection of the assemblies and detection of contig groups potentially derived from non-target organisms [52]. The contamination-filtered assemblies were further processed by removing contigs shorter than 200 bp. The resulting genome FASTA files were used for annotation and comparative genomic analyses. Genome annotation was performed with funannotate v1.8.17 (https://github.com/nextgenusfs/funannotate, accessed on 10 April 2026). Repetitive regions were identified with RepeatMasker v4.2.3 (https://www.repeatmasker.org, accessed on 10 April 2026) using Dfam repeat libraries [53]. Gene prediction and annotation were carried out with the funannotate predict and annotate workflows. Assembly completeness was assessed with BUSCO v5.8.3 in genome mode using the sordariomycetes_odb10 lineage dataset (n = 3817). Completeness of the final predicted protein sets was assessed separately in protein mode using the same lineage dataset [54]. Functional annotation included eggNOG-mapper v2.1.13 with DIAMOND v2.0.15 searches against the eggNOG database [55,56] and InterProScan v5.77-108.0 [57]. The annotated genome assemblies used in this study are provided in Supplementary File S1. The *C. nigrum* C21KSPeF19 genome was deposited in GenBank under WGS accession JBZXJN000000000 (BioProject PRJNA1479150). The *C. coccodes* Cc17-M4 and Cc46-Lip3 genome submissions are registered under BioProjects PRJNA1460358 and PRJNA1460360, respectively; their GenBank accession numbers were pending at the time of manuscript submission.

Secreted proteins and candidate effectors were predicted from the protein sequences generated by the funannotate gene-prediction workflow. Signal peptides were predicted with SignalP v6.0h [58], subcellular targeting was assessed with TargetP v2.0 [59], and transmembrane topology was analyzed with DeepTMHMM v1.0 (https://services.healthtech.dtu.dk/services/DeepTMHMM-1.0/, accessed on 11 April 2026). SignalP and TargetP predictions were retained as complementary protein annotations and were not used to define the final secretome. The predicted secretome was defined as proteins assigned to the SP class by DeepTMHMM; proteins assigned to other DeepTMHMM classes were excluded. The corresponding protein sequences were extracted from each predicted proteome and used as input for EffectorP v3.0 [60] in fungal mode (-f) to predict candidate effectors. Putative CYP450-like proteins were identified from InterProScan results using the InterPro accessions IPR001128 (Cytochrome P450), IPR036396 (Cytochrome P450 superfamily), and IPR002401 (Cytochrome P450, E-class, group I), together with records containing the term “cytochrome P450” in domain or annotation descriptions.

CAZyme annotation was performed with run_dbCAN/dbCAN v5.2.9 [61]. Proteins assigned to a CAZyme family by at least one dbCAN component were retained as the broad CAZyme inventory, whereas proteins supported by at least two dbCAN tools were considered the high-confidence CAZyme set. Predicted secreted CAZymes were identified by intersecting the broad CAZyme-family-assigned set with the DeepTMHMM SP secretome. CAZyme annotations were also intersected with EffectorP predictions to identify effector-associated CAZymes.

Secondary-metabolite biosynthetic gene clusters were predicted with antiSMASH v8.0.4 in fungal mode [62]. GenBank files from the final genome annotations were used as input. antiSMASH was run with gene finding disabled to preserve the original annotation coordinates. Predicted regions were summarized by product class. Each antiSMASH region was counted once as a predicted genomic region, whereas product-class labels were summarized separately because a single region could contain multiple product designations.

Pairwise genome-wide nucleotide similarity was assessed with FastANI v1.34 in all-versus-all mode [36]. Filtered genome FASTA files for *C. coccodes* 17-M4, *C. coccodes* 46-Lip3, and *C. nigrum* C21KSPeF19 were used as both query and reference genomes. Directional ANI values were converted into a 3 × 3 matrix. For each non-identical genome pair, a symmetric mean ANI value was calculated as the arithmetic mean of the two reciprocal fastANI estimates. The mapped/total fragment counts reported by fastANI were retained for interpretation of pairwise comparisons.

Orthogroup inference was performed with OrthoFinder v3.1.5 using DIAMOND v2.2.1 for protein sequence searches [56,63], and the predicted proteomes of *C. coccodes* Cc17-M4, *C. coccodes* Cc46-Lip3, and *C. nigrum* C21KSPeF19 were used as input. Orthogroups were classified by their presence or absence in the three genomes. Orthogroups shared by both *C. coccodes* genomes and absent from *C. nigrum* C21KSPeF19, and orthogroups specific to C21KSPeF19, were extracted and annotated using funannotate product names, eggNOG and InterProScan results, secretome predictions, EffectorP output, dbCAN CAZyme assignments, and CYP450-like protein screening.

### 4.5. Morphological and Morphometric Characterization

Mycelial agar disks (7.5 mm diameter) were removed from the actively growing edge of seven-day cultures of *C. coccodes* and transferred to PDA to assess the morphology of colonies, conidia and sclerotia. The plates were incubated at 25 °C in the dark, and after 7 days, colony morphology and sclerotial distribution were assessed visually by a single observer. Stability of morphology groups across repeated subcultures was not formally assessed in this study. The categories were used for descriptive classification and selection of isolates for morphometry, not as quantitative measurements or validated stable traits. For microscopic analysis, twenty-two isolates representing four different colony morphological groups were taken. Plates were flooded with SDW, and conidia and sclerotia were scraped with a loop to describe conidia and sclerotia morphology. The length and width of at least 30 conidia and 50 sclerotia were measured using ImageJ 1.54g (National Institutes of Health, Bethesda, MD, USA).

The physiological characterization included examining the growth rate of isolates on PDA at 25 °C. Each of the 68 isolates was represented by three separately inoculated plates (N = 204). Colony diameter growth rate on PDA at 25 °C was determined from measurements on days 5 and 7. Two perpendicular colony diameters were measured for each plate at each time point and averaged. Growth rate was calculated as (D_7_ − D_5_)/(7 − 5) and expressed in mm/day. Subtraction of the initial inoculum-plug diameter was unnecessary because the diameter cancels out when the difference between the two measurements is calculated. The effect of temperature on colony growth rate of isolates was assessed according to [64] with modifications. Briefly, three isolates from southern regions (Krasnodar, Astrakhan) and three from northern regions (Moscow, Tver, Nizhniy Novgorod) were incubated at 10 °C, 15 °C, 20 °C, 25 °C, 30 °C and 35 °C, and colony diameter was measured after 5 and 7 days of incubation. The results were analyzed by two-way ANOVA. Each isolate × temperature combination was represented by three separately inoculated Petri dishes in one experimental run (six isolates × six temperatures × three plates; N = 108). Because incubation at 25 °C produced the highest overall mean growth rate across isolates, growth at each temperature was expressed relative to the corresponding mean growth rate at 25 °C for visualization.

The effect of culture media on growth rate was assessed by cultivating 17 selected different geographical isolates on six culture media: Asthana & Hawker agar, Czapek–Dox agar, Sabouraud dextrose agar (SDA), corn meal agar (CMA), rye agar and potato dextrose agar (PDA) (Table 3).

Mycelium agar disks (7.5 mm diameter) were taken from the actively growing margin of 5-to-7-day-old colonies and placed in the center of the test plates. Plates were incubated for 7 days, and the growth rate was determined as described before. For each isolate × medium combination, three separate 90 mm Petri dishes were inoculated simultaneously and incubated at 25 °C in the dark. Plate positions within the incubator were randomized. Colony diameters were measured on days 5 and 7, and growth rates were calculated as described above.

### 4.6. Pathogenicity and Aggressiveness Assay

Pathogenicity and aggressiveness were assessed using a potato tuber slice assay with modifications. Potato tubers of cv. Arrow were washed, surface-sterilized for 10 min in 10% (*v*/*v*) aqueous solution of the Domestos product described in Section 4.1, rinsed three times with sterile distilled water, and cut into slices 5–7 cm in diameter and approximately 1 cm thick using a sterile knife (Huaiyin Medical Instruments Co., Ltd., Huaian, China). A 7.5 mm mycelium disk taken from the actively growing margin of a 7-day-old culture was placed in the center of each tuber slice. Each isolate was tested on three tuber slices, with the three slices originating from three different tubers. Three additional slices inoculated with sterile PDA plugs served as negative controls. The slices were incubated in moist chambers at 25 °C in the dark for 15 days. Tubers of cv. Arrow used in this study were obtained from the field station of Russian State Agrarian University—Moscow Timiryazev Agricultural Academy (RSAU–MTAA). All tubers were harvested from a single production batch in September 2025, were of similar physiological age, and were stored at 3 °C for 2 months prior to the experiment. Lesion diameter was measured in two perpendicular directions using a digital caliper ADA Mechanic 150 PRO (ADA INSTRUMENTS Co LTD., Shenzhen, China). and averaged for each replicate. To confirm recovery of the inoculated fungus from symptomatic tissue, fragments from the lesion margin were transferred onto PDA, and the re-isolated fungi were identified based on colony morphology under a Stemi 508 stereomicroscope and conidial morphology under a Primostar 3 light microscope (Zeiss, Oberkochen, Germany); the identity of re-isolates was confirmed by sequencing *act* and *cal* loci. The experiment included 3 biological replicates per isolate; each biological replicate consisted of one tuber slice obtained from a separate potato tuber. Tuber slices were randomly assigned to isolates, and inoculated slices were randomly positioned within the incubation chambers. Because inoculation was performed on freshly cut tuber tissue, the assay was intended primarily for comparative evaluation of aggressiveness rather than simulation of natural infection.

### 4.7. Statistical Analysis and Visualization

Obtained data on growth characteristics (growth rate on PDA and other culture media, lesion diameter on potato slices) were analyzed in Statistica v.10 (TIBCO, Palo-Alto, CA, USA) by one-way ANOVA followed by post hoc Tukey’s HSD test.

A correlation between the growth rate on PDA and lesion diameter on potato tuber slices was evaluated using Spearman’s correlation coefficient (*ρ*).

The influence of the Isolate, Medium, and interaction factors on colony growth rate was assessed using a two-way ANOVA. The effect size of each factor was determined using partial *η*^2^.

The influence of Isolate, Temperature and their interaction factors on colony growth was assessed using two-way ANOVA. Differences were considered statistically significant at *p* < 0.05.

For all parametric tests (one-way and two-way ANOVA), the assumptions of normality of residuals (Shapiro–Wilk test) and homogeneity of variances (Brown–Forsythe test, Levene’s test) were checked. Residuals of the two-way ANOVA models were also visually inspected using Q–Q plots. The complete raw replicate data for experiments, including lesion diameters, growth rates on PDA, growth on six nutrient media, and temperature response, are provided in Supplementary Table S8.

Visualization of the data was performed in GraphPad Prism 10.6.0 (GraphPad Software, Boston, MA, USA).

## 5. Conclusions

In this study, we provided a comprehensive characterization of 68 *Colletotrichum coccodes* isolates recovered from potato in Russia and Belarus. The collection displayed substantial phenotypic diversity, including variation in colony morphology, sclerotial distribution, mycelial growth rate, culture-medium preference, and aggressiveness in a wounded detached tuber slice assay. Despite this phenotypic variation, multilocus sequence analysis revealed only five haplotypes, with limited nucleotide polymorphism, and whole-genome comparison of two *C. coccodes* isolates confirmed high genomic similarity, suggesting that the observed phenotypic diversity is not reflected in the small set of loci examined.

## Figures and Tables

**Table 1:** Morphometric characteristics of conidia and sclerotia of representative *Colletotrichum coccodes* isolates.

Morphology Group	Isolate ID	Conidial Length, µm	Conidial Width, µm	Sclerotial Length, µm	Sclerotial Width, µm
A	Cc12-T1	16.1 ± 0.2	4.2 ± 0.6	352 ± 76	271 ± 57
A	Cc51-BLR1	11.7 ± 2.4	4.7 ± 0.9	149 ± 32	130 ± 27
A	Cc15-As1	16.0 ± 2.1	4.2 ± 0.7	256 ± 55	202 ± 29
A	Cc21-Kr2	20.7 ± 2.6	3.8 ± 0.4	97 ± 27	73 ± 8
A	Cc29-M11	11.7 ± 2.3	5.1 ± 0.9	150 ± 41	86 ± 16
B	Cc1-M1	18.6 ± 3.1	4.3 ± 0.6	247 ± 42	218 ± 37
B	Cc17-M4	17.3 ± 1.9	4.2 ± 0.8	226 ± 55	195 ± 28
B	Cc9-N4	18.5 ± 2.6	4.5 ± 0.6	288 ± 76	256 ± 53
B	Cc14-St1	16.3 ± 2.3	4.6 ± 0.8	249 ± 59	194 ± 31
B	Cc11-N6	14.9 ± 4.0	4.2 ± 0.7	295 ± 63	206 ± 40
B	Cc24-M6	13.5 ± 3.9	5.5 ± 1.2	116 ± 21	93 ± 16
C	Cc13-Kr1	19.2 ± 2.1	3.5 ± 0.7	278 ± 53	199 ± 30
C	Cc7-N2	19.9 ± 2.1	6.0 ± 0.8	106 ± 22	79 ± 15
C	Cc40-M14	12.2 ± 2.6	4.8 ± 1.2	118 ± 24	104 ± 16
C	Cc48-Tul1	14.3 ± 2.5	5.2 ± 1.0	129 ± 17	104 ± 16
C	Cc57-Tvr1	14.6 ± 2.9	6.3 ± 1.4	209 ± 71	136 ± 39
D	Cc4-P1	16.2 ± 2.1	5.7 ± 1.0	303 ± 76	213 ± 31
D	Cc6-N1	17.1 ± 2.3	5.8 ± 0.5	349 ± 88	253 ± 39
D	Cc18-M5	15.9 ± 2.8	4.2 ± 0.7	303 ± 73	207 ± 31
D	Cc2-M2	18.0 ± 2.9	5.1 ± 0.5	243 ± 69	204 ± 48
D	Cc3-M3	15.3 ± 3.3	5.8 ± 1.0	375 ± 93	290 ± 42
D	Cc8-N3	13.7 ± 2.7	5.6 ± 0.8	287 ± 49	286 ± 88

Note: Values are means ± SD based on measurements of at least 30 conidia and 50 sclerotia per isolate.

**Table 2:** Primers used for PCR amplification and sequencing.

Locus	Primer Name	Primer Sequence (5′–3′)	Annealing Temperature, °C	Reference
ITS	ITS1	TCCGTAGGTGAACCTGCGG	55	[40]
	ITS4	TCCTCCGCTTATTGATATGC		
*act*	ACT-512F	ATGTGCAAGGCCGGTTTCGC	67	[41]
	ACT-783R	TACGAGTCCTTCTGGCCCAT		
*cal*	CL1C	GAATTCAAGGAGGCCTTCTC	57	[43]
	CL2C	CTTCTGCATCATGAGCTGGAC		
*his3*	CYLH3F	AGGTCCACTGGTGGCAAG	52	[44]
	CYLH3R	AGCTGGATGTCCTTGGACTG		

**Table 3:** Culture media used to evaluate the effect on colony growth rate.

Culture Media	Composition (per 1 L of Distilled Water)	Reference
Asthana & Hawker agar	Glucose 5 g, potassium nitrate 3.5 g, potassium dihydrogenphosphate 1.75 g, magnesium sulfate 0.75 g, agar 15 g	[65]
Czapek–Dox agar	Sodium nitrate 2 g, potassium hydrogen phosphate 1 g, magnesium sulfate 0.5 g, potassium chloride 0.5 g, ferrous sulphate 0.01 g, sucrose 30 g, agar 15 g	[65]
Sabouraud dextrose agar	Dextrose 40 g, yeast peptone 10 g, agar 15 g	[66]
Corn meal agar	Corn meal 30 g, agar 15 g	[65]
Rye agar	Rye grain 60 g, dextrose 20 g, agar 15 g	[67]
Potato dextrose agar	Potato 200 g, dextrose 20 g, agar 15 g	[65]

## Data Availability

Raw paired-end genome sequencing reads generated in this study are available in the NCBI Sequence Read Archive under accession numbers SRR40413653 (*C. coccodes* Cc17-M4), SRR40412965 (*C. coccodes* Cc46-Lip3), and SRR40413900 (*C. nigrum* C21KSPeF19). The annotated genome assembly of *C. nigrum* C21KSPeF19 is available in NCBI GenBank under WGS accession JBZXJN000000000 (BioProject PRJNA1479150). The genome assemblies of *C. coccodes* Cc17-M4 and Cc46-Lip3 were submitted to NCBI under BioProjects PRJNA1460358 and PRJNA1460360, respectively; their final WGS accession numbers remained pending at the time of revision. The BioSample IDs are available in NCBI: SAMN57591514 (Cc17-M4), SAMN57591647 (Cc46-Lip3) and SAMN60943264 (C21KSPeF19). Annotated GenBank-format assemblies of all three genomes are additionally provided in Supplementary File S1. The ITS, *cal*, *his3*, and *act* sequences generated in this study are available in GenBank under the accession numbers listed in Supplementary Table S1.

## Supplementary Materials

The following supporting information can be downloaded at: https://www.mdpi.com/article/10.3390/plants15182853/s1, Table S1. Origin, isolation source, GenBank accession numbers, and colony characteristics of the *Colletotrichum coccodes* isolates examined in this study. Table S2. Growth rates of 17 *Colletotrichum coccodes* isolates on six culture media. Table S3. SNP haplotypes of *Colletotrichum coccodes* isolates based on MLST loci. The table summarizes five SNP haplotypes identified among 68 isolates using five variable nucleotide positions in *cal*, *his3*, and ITS loci. The *act* locus was included in the four-locus concatenated MLST alignment but was invariant among the analyzed isolates. SNP coordinates are given as locus:position and are calculated relative to the ungapped sequence of the corresponding locus in the *C. coccodes* CBS 369.75 reference strain. Isolates with identical nucleotide states across all SNP positions were assigned to the same SNP haplotype. Table S4. Genome sequencing, assembly, and completeness statistics for the three sequenced *Colletotrichum* genomes. Table S5. Reciprocal FastANI comparisons among the three sequenced *Colletotrichum* genomes. ANI values, mapped and total query-fragment counts, and mapped query-fragment fractions are reported for each directional comparison. The mapped query-fragment fraction was calculated as the number of mapped fragments divided by the total number of query fragments × 100. Table S6. Gene-level annotation of 407 candidate *Colletotrichum coccodes*-associated orthogroups identified in both sequenced *C. coccodes* isolates and absent from the *C. nigrum* C21KSPeF19 OrthoFinder assignment. Table S7. Reference strains and GenBank accession numbers of sequences used in the species-level phylogenetic analysis. Table S8. Replicate data for *C. coccodes* isolates: growth rates and lesion diameters. File S1. Annotated GenBank-format genome assemblies of *Colletotrichum coccodes* Cc17-M4 and Cc46-Lip3 and *Colletotrichum nigrum* C21KSPeF19. The file is provided to ensure access to the genome assemblies and annotations during the NCBI processing of the two *C. coccodes* submissions. The *C. nigrum* C21KSPeF19 genome is available under WGS accession JBZXJN000000000 (BioProject PRJNA1479150), whereas final WGS accession numbers for *C. coccodes* Cc17-M4 (BioProject PRJNA1460358) and Cc46-Lip3 (BioProject PRJNA1460360) were still pending at the time of revision.

## Abbreviations

The following abbreviations are used in this manuscript:

AFLP	Amplified-Fragment Length Polymorphism
ANI	Average Nucleotide Identity
BUSCO	Benchmarking Universal Single-Copy Orthologs
CAZyme	Carbohydrate-Active Enzyme
CDS	Coding DNA Sequence
CMA	Corn Meal Agar
DNA	Deoxyribonucleic Acid
ITS	Internal Transcribed Spacer
MLST	Multilocus Sequence Typing
PDA	Potato Dextrose Agar
SDA	Sabouraud Dextrose Agar
SDW	Sterile Distilled Water
SNP	Single-Nucleotide Polymorphism
VCG	Vegetative Compatibility Group
