# Supplementary material for: Morphological, Physiological, Pathogenic, and Molecular Characterization of Colletotrichum coccodes Isolates Associated with Potato Black Dot
Source: Plants (Basel). 2026 Sep 18;15(18):2853. doi: 10.3390/plants15182853 (PMC13610318; doi:10.3390/plants15182853)
Supplement: Supplementary file 1 [file plants-15-02853-s001.zip › Supplementary_Table_S1.pdf]

№	Isolate ID	Year of isolation	Region (or country)	District	Source of isolation	Cultivar of potato host	GenBank accession numbers				Colony morphology group	Colony color
							ITS	ACT	HIS3	CAL		
1	Cc1-M1	2023	Moscow	Kolomensky	tuber	Gala	PZ577964	PZ684186	PZ595096	PZ595025	B	White
2	Cc2-M2	2023	Moscow	Dmitrovsky	tuber	Primabelle	PZ577965	PZ684187	PZ595097	PZ595026	D	White
3	Cc3-M3	2023	Moscow	Dmitrovsky	tuber	Gala	PZ577966	PZ684188	PZ595098	PZ595027	D	White
4	Cc4-P1	2023	Penza	N/A	tuber	Gala	PZ577967	PZ684189	PZ595099	PZ595028	D	White
5	Cc5-P2	2023	Penza	N/A	tuber	Gala	PZ577968	PZ684190	PZ595100	PZ595029	B	White
6	Cc6-N1	2023	Nizhny Novgorod	N/A	tuber	N/A	PZ577969	PZ684191	PZ595101	PZ595030	D	White
7	Cc7-N2	2023	Nizhny Novgorod	N/A	tuber	Colomba	PZ577970	PZ684192	PZ595102	PZ595031	C	White
8	Cc8-N3	2024	Nizhny Novgorod	Arzamas	tuber	Gala	PZ577971	PZ684193	PZ595103	PZ595032	D	White
9	Cc9-N4	2024	Nizhny Novgorod	Arzamas	tuber	Gala	PZ577972	PZ684194	PZ595104	PZ595033	B	White
10	Cc10-N5	2024	Nizhny Novgorod	N/A	tuber	Belmonda	PZ577973	PZ684195	PZ595105	PZ595034	D	White
11	Cc11-N6	2024	Nizhny Novgorod	N/A	tuber	Red Scarlett	PZ577974	PZ684196	PZ595106	PZ595035	B	White
12	Cc12-T1	2024	Tomsk	N/A	tuber	Gala	PZ577975	PZ684197	PZ595107	PZ595036	A	White
13	Cc13-Kr1	2024	Krasnodar	N/A	tuber	Colomba	PZ577976	PZ684198	PZ595108	PZ595037	C	White
14	Cc14-St1	2024	Stavropol	Ipatovsky	tuber	VR-808	PZ577977	PZ684199	PZ595109	PZ595038	B	White
15	Cc15-As1	2024	Astrakhan	Limansky	tuber	Arizona	PZ577978	PZ684200	PZ595110	PZ595039	A	White
16	Cc16-Br1	2024	Bryansk	Komarinsky	tuber	N/A	PZ577979	PZ684201	PZ595111	PZ595040	C	Yellow
17	Cc17-M4	2024	Moscow	Moscow, station of plant protection of RSAU-MTAA	tuber	N/A	PZ577980	PZ684202	PZ595112	PZ595041	B	White
18	Cc18-M5	2024	Moscow	N/A	tuber	N/A	PZ577981	PZ684203	PZ595113	PZ595042	D	White
19	Cc19-Bel1	2024	Belgorod	Prokhorovsky	tuber	Gala	PZ577982	PZ684204	PZ595114	PZ595043	D	White
20	Cc20-Tat1	2024	Tatarstan	Visokogorsky	tuber	Gala	PZ577983	PZ684205	PZ595115	PZ595044	D	White
21	Cc21-Kr2	2024	Krasnodar	Otrasnensky	tuber	VR-808	PZ577984	PZ684206	PZ595116	PZ595045	A	White
22	Cc22-Lip1	2024	Lipetsk	Khlevensky	tuber	Gala	PZ577985	PZ684207	PZ595117	PZ595046	B	White
23	Cc23-Lip2	2024	Lipetsk	Khlevensky	tuber	Prime	PZ577986	PZ684208	PZ595118	PZ595047	B	White
24	Cc24-M6	2024	Moscow	N/A	stem	N/A	PZ577987	PZ684209	PZ595119	PZ595048	B	White

25	Cc25-M7	2024	Moscow	Kolomna	stem	Sagitta	PZ577988	PZ684210	PZ595120	PZ595049	D	White
26	Cc26-M8	2024	Moscow	Kolomna	stem	Sagitta	PZ577989	PZ684211	PZ595121	PZ595050	D	White
27	Cc27-M9	2024	Moscow	Kolomna	stem	Sagitta	PZ577990	PZ684212	PZ595122	PZ595051	D	White
28	Cc28-M10	2024	Moscow	Kolomna	stem	Sagitta	PZ577991	PZ684213	PZ595123	PZ595052	C	White
29	Cc29-M11	2024	Moscow	Kolomna	stem	Sagitta	PZ577992	PZ684214	PZ595124	PZ595053	A	White
30	Cc30-Or1	2024	Oryol	Mtsensk	stem	N/A	PZ577993	PZ684215	PZ595125	PZ595054	B	White
31	Cc31-Or2	2024	Oryol	Mtsensk	stem	N/A	PZ577994	PZ684216	PZ595126	PZ595055	D	White
32	Cc32-Or3	2024	Oryol	Mtsensk	stem	N/A	PZ577995	PZ684217	PZ595127	PZ595056	C	White
33	Cc33-Or4	2024	Oryol	Mtsensk	stem	N/A	PZ577996	PZ684218	PZ595128	PZ595057	B	White
34	Cc34-Or5	2024	Oryol	Mtsensk	stem	N/A	PZ577997	PZ684219	PZ595129	PZ595058	B	White
35	Cc35-Or6	2024	Oryol	Mtsensk	stem	N/A	PZ577998	PZ684220	PZ595130	PZ595059	C	White
36	Cc36-Or7	2024	Oryol	Mtsensk	stem	N/A	PZ577999	PZ684221	PZ595131	PZ595060	B	White
37	Cc37-Or8	2024	Oryol	Mtsensk	tuber	N/A	PZ578000	PZ684222	PZ595132	PZ595061	D	White
38	Cc38-M12	2025	Moscow	N/A	tuber	N/A	PZ578001	PZ684223	PZ595133	PZ595062	C	White
39	Cc39-M13	2025	Moscow	N/A	tuber	N/A	PZ578002	PZ684224	PZ595134	PZ595063	C	White
40	Cc40-M14	2025	Moscow	N/A	tuber	N/A	PZ578003	PZ684225	PZ595135	PZ595064	C	White
41	Cc41-M15	2025	Moscow	N/A	tuber	N/A	PZ578004	PZ684226	PZ595136	PZ595065	C	White
42	Cc42-M16	2025	Moscow	N/A	tuber	N/A	PZ578005	PZ684227	PZ595137	PZ595066	B	Yellow
43	Cc43-M17	2025	Moscow	N/A	tuber	N/A	PZ578006	PZ684228	PZ595138	PZ595067	C	Yellow
44	Cc44-M18	2025	Moscow	N/A	tuber	N/A	PZ578007	PZ684229	PZ595139	PZ595068	C	Yellow
45	Cc45-M19	2025	Moscow	N/A	tuber	N/A	PZ578008	PZ684230	PZ595140	PZ595069	B	White
46	Cc46-Lip3	2025	Lipetsk	Usmansky	tuber	N/A	PZ578009	PZ684231	PZ595141	PZ595070	D	White
47	Cc47-Lip4	2025	Lipetsk	Usmansky	tuber	N/A	PZ578010	PZ684232	PZ595142	PZ595071	D	Yellow
48	Cc48-Tul1	2025	Tula	N/A	tuber	N/A	PZ578011	PZ684233	PZ595143	PZ595072	C	White
49	Cc49-Tul2	2025	Tula	N/A	tuber	N/A	PZ578012	PZ684234	PZ595144	PZ595073	B	White
50	Cc50-St2	2025	Stavropol	N/A	tuber	N/A	PZ578013	PZ684235	PZ595145	PZ595074	B	White
51	Cc51-BLR1	2025	Republic of Belarus	N/A	tuber	N/A	PZ578014	PZ684236	PZ595146	PZ595075	A	White
52	Cc52-Br2	2026	Bryansk	Starodubsky	tuber	VR-808	PZ578015	PZ684237	PZ595147	PZ595076	D	Yellow
53	Cc53-Chlb1	2026	Chelyabinsk	N/A	tuber	Arizona	PZ578016	PZ684238	PZ595148	PZ595077	B	White

54	Cc54-Bel2	2026	Belgorod	Chernyansky	tuber	Krone	PZ578017	PZ684239	PZ595149	PZ595078	A	White
55	Cc55-Bel3	2026	Belgorod	Chernyansky	tuber	Gala	PZ578018	PZ684240	PZ595150	PZ595079	B	White
56	Cc56-Bel4	2026	Belgorod	Chernyansky	tuber	Challenger	PZ578019	PZ684241	PZ595151	PZ595080	C	White
57	Cc57-Tvr1	2026	Tver	Staritsky	tuber	Gala	PZ578020	PZ684242	PZ595152	PZ595081	C	White
58	Cc58-Tvr2	2026	Tver	Staritsky	tuber	Gala	PZ578021	PZ684243	PZ595153	PZ595082	C	White
59	Cc59-M20	2026	Moscow	Dmitrovsky	tuber	Colomba	PZ578022	PZ684244	PZ595154	PZ595083	D	White
60	Cc60-N7	2026	Nizhny Novgorod	Arzamas	tuber	Gala	PZ578023	PZ684245	PZ595155	PZ595084	D	Yellow
61	Cc61-M21	2026	Moscow	Ozyory Urban District	tuber	Carmen	PZ685581	PZ684246	PZ595156	PZ595085	C	White
62	Cc62-M22	2026	Moscow	N/A	tuber	N/A	PZ578024	PZ684247	PZ595157	PZ595086	B	White
63	Cc63-BLR2	2026	Republic of Belarus	N/A	tuber	Soraya	PZ578025	PZ684248	PZ595158	PZ595087	C	White
64	Cc64-N8	2026	Nizhny Novgorod	Arzamas	tuber	Gala	PZ578026	PZ684249	PZ595159	PZ595088	C	White
65	Cc65-Bshk1	2026	Bashkortostan	N/A	tuber	Memphis	PZ578027	PZ684250	PZ595160	PZ595089	D	White
66	Cc66-BLR3	2026	Republic of Belarus	N/A	tuber	Mia	PZ578028	PZ684251	PZ595161	PZ595090	B	White
67	Cc67-St3	2026	Stavropol	Kochubeevsky	tuber	VR-808	PZ578029	PZ684252	PZ595162	PZ595091	B	White
68	Cc68-VNF	2026	Moscow	Odintsovky	tuber	N/A	PZ578030	PZ684253	PZ595163	PZ595092	B	Yellow
