# Supplementary material for: Morphological, Physiological, Pathogenic, and Molecular Characterization of Colletotrichum coccodes Isolates Associated with Potato Black Dot
Source: Plants (Basel). 2026 Sep 18;15(18):2853. doi: 10.3390/plants15182853 (PMC13610318; doi:10.3390/plants15182853)
Supplement: Supplementary file 1 [file plants-15-02853-s001.zip › Supplementary_Table_S2.pdf]

№	Isolate	Growth rate, mm/day on different nutrient medium					
		Asthana & Hawker agar	Czapek-Dox agar	Sabouraud dextrose agar	Corn meal agar	Rye agar	Potato dextrose agar
1	Cc1-M1	7.6 ± 0.25	7.1 ± 0.08	10.2 ± 0.60	8.6 ± 0.06	9.2 ± 0.22	9.9 ± 0.83
2	Cc2-M2	7.8 ± 0.20	8.1 ± 0.11	11.1 ± 0.20	9.1 ± 0.13	8.8 ± 1.02	10.2 ± 0.15
3	Cc3-M3	7.9 ± 0.22	7.2 ± 0.78	10.9 ± 0.25	9.2 ± 0.82	8.8 ± 0.26	9.1 ± 1.44
4	Cc4-P1	6.4 ± 2.97	6.5 ± 2.6	10.5 ± 0.69	8.5 ± 1.43	7.5 ± 1.62	9.4 ± 0.72
5	Cc5-P2	7.7 ± 0.25	7.8 ± 0.20	11.2 ± 0.13	9.0 ± 0.82	9.5 ± 0.12	9.7 ± 0.31
6	Cc6-N1	7.5 ± 0.06	8.2 ± 1.35	10.9 ± 0.59	8.9 ± 0.41	9.9 ± 0.03	10.3 ± 0.47
7	Cc7-N2	7.2 ± 0.58	9.5 ± 0.36	10.9 ± 0.60	8.6 ± 0.22	9.2 ± 0.50	9.7 ± 0.31
8	Cc8-N3	8.3 ± 0.70	9.6 ± 0.43	9.4 ± 0.46	8.6 ± 0.19	9.3 ± 0.03	9.5 ± 0.31
9	Cc9-N4	8.2 ± 0.50	9.6 ± 0.20	9.4 ± 0.54	8.7 ± 0.29	9.2 ± 0.34	9.0 ± 0.35
10	Cc11-N6	7.9 ± 0.29	9.8 ± 0.56	10.9 ± 1.04	8.9 ± 1.04	9.3 ± 0.33	9.8 ± 0.67
11	Cc12-T1	8.2 ± 0.32	11.1 ± 0.70	11.0 ± 0.25	8.8 ± 0.16	8.4 ± 0.76	9.9 ± 0.80
12	Cc13-Kr1	8.1 ± 0.47	10.4 ± 0.67	9.9 ± 1.2	8.0 ± 0.1	8.5 ± 0.31	9.9 ± 0.80
13	Cc14-St1	6.8 ± 0.31	9.1 ± 0.50	9.8 ± 0.27	8.4 ± 0.09	8.4 ± 1.14	8.9 ± 0.56
14	Cc15-As1	7.6 ± 0.42	10.9 ± 0.36	9.6 ± 0.46	8.1 ± 0.19	7.9 ± 0.62	9.1 ± 0.1
15	Cc17-M4	9.1 ± 0.58	10.9 ± 0.43	10.6 ± 0.52	8.8 ± 0.1	9.7 ± 0.58	10.6 ± 0.14
16	Cc18-M5	7.8 ± 0.5	8.9 ± 1.1	10.3 ± 0.5	9.0 ± 0.4	8.6 ± 0.5	9.5 ± 0.4
17	Cc19-Bel1	7.2 ± 0.2	9.3 ± 0.2	9.7 ± 0.5	8.4 ± 0.2	8.3 ± 0.5	10.3 ± 0.7
Mean		7.8 ± 0.6	9.2 ± 1.3	10.3 ± 0.8	8.7 ± 0.5	8.8 ± 0.8	9.7 ± 0.7

Note: All values represent the mean ± SD of three replicate Petri dishes.
