# Supplementary material for: Morphological, Physiological, Pathogenic, and Molecular Characterization of Colletotrichum coccodes Isolates Associated with Potato Black Dot
Source: Plants (Basel). 2026 Sep 18;15(18):2853. doi: 10.3390/plants15182853 (PMC13610318; doi:10.3390/plants15182853)
Supplement: Supplementary file 1 [file plants-15-02853-s001.zip › Supplementary_Table_S3.pdf]

SNP haplotype	Number of isolates	CAL:37	CAL:117	HIS3:230	HIS3:360	ITS:449	Isolates
H1	16	C	G	G	C	C	Cc4-P1, Cc17-M4, Cc18-M5, Cc19-Bel1, Cc34-Or5, Cc37-Or8, Cc41-M15, Cc49-Tul2, Cc50-St2, Cc52-Br2, Cc56-Bel4, Cc57-Tvr1, Cc58-Tvr2, Cc61-M21, Cc66-BLR3, Cc67-St3
H2	19	C	G	G	T	C	Cc2-M2, Cc3-M3, Cc5-P2, Cc6-N1, Cc10-N5, Cc11-N6, Cc12-T1, Cc20-Tat1, Cc21-Kr2, Cc22-Lip1, Cc23-Lip2, Cc24-M6, Cc29-M11, Cc45-M19, Cc51-BLR1, Cc53-Chlb1, Cc54-Bel2, Cc62-M22, Cc63-BLR2
H3	1	C	G	T	C	C	Cc7-N2
H4	7	T	C	G	C	A	Cc27-M9, Cc38-M12, Cc39-M13, Cc40-M14, Cc46-Lip3, Cc59-M20, Cc64-N8
H5	25	T	C	G	C	C	Cc1-M1, Cc8-N3, Cc9-N4, Cc13-Kr1, Cc14-St1, Cc15-As1, Cc16-Br1, Cc25-M7, Cc26-M8, Cc28-M10, Cc30-Or1, Cc31-Or2, Cc32-Or3, Cc33-Or4, Cc35-Or6, Cc36-Or7, Cc42-M16, Cc43-M17, Cc44-M18, Cc47-Lip4, Cc48-Tul1, Cc55-Bel3, Cc60-N7, Cc65-Bshk1, Cc68-VNF
